# Can Trauma-Informed Yoga Center Intersectional Feminist Praxis? The Case of a UK Community Yoga Organization

**DOI:** 10.1177/10778012241275690

**Published:** 2024-09-10

**Authors:** Evanthia Triantafyllidou, Megan Cowles, Leonie Harvey-Rolfe

**Affiliations:** 1 Centre for Gender and Violence Research, School for Policy Studies, University of Bristol, Bristol, UK; 2 Bristol Yoga Roots Project, Bristol, UK

**Keywords:** trauma-informed, embodiment, feminist, yoga, healing

## Abstract

This article discusses the tensions around trauma-informed narratives and mind-body practices, which may obscure social inequalities. We present the evaluation of community yoga programs and explore how trauma-informed yoga can be part of the healing process of women subject to interlocking systems of oppression. The study showed how the sociocultural location of participants shaped their engagement with normative yoga discourses and practices. Yoga was perceived as a practice that improved the sense of healing and well-being, and created relational spaces during COVID-19. The article also discusses the value of embodied self-inquiry as an intersectional feminist tool for researchers and practitioners.

## Introduction

The increasing popularity of trauma-informed approaches alongside the growing evidence on the centrality of the body in the traumatic experience, led to the development of trauma-informed yoga practices. At the same time, the need for a move away from the commercialization of yoga within the mainstream yoga spaces led to the creation of community yoga projects. This article aims to examine if and how trauma-informed yoga programs can be underpinned by an intersectional feminist ethos. These questions have emerged due to the ongoing tensions around the depoliticized agenda of trauma and yoga “industries.” Feminists have been arguing against the growing professionalization of trauma (trauma industry)—within the violence against women services—for pathologizing women and sidelining the societal causes of violence (Brown, 2004; Gilfus, 1999; Tseris, 2013, 2017; Vera-Gray, 2020). Feminist contribution to the trauma debate has pushed for a more politicized lens with Burstow (2003) pointing to a "trauma continuum" (p.1310), while Brown (1995) and Herman (1997) discussed the insidious nature of trauma due to domestic and sexual violence. At the same time, decolonial and yoga scholars have criticized the yoga consumerist world (yoga industry) for privileging the pursuit of self-care, promoting neoliberal agendas of self- governance, and effacing social inequalities (Batacharya, 2018; Jain, 2020; Lucia, 2020). Additionally, yoga scholars and practitioners (Jain, 2020; Wildcroft & McAtee, 2024) have critically examined the underlying class and race dynamics within the modern yoga spaces, and explored the possibilities offered by community yoga projects which attempt to counteract the existing inequalities within the yoga industry. Community yoga projects aim to bring in a more politicized lens to yoga provision and create communities of practice, where personal healing is interwoven with systemic change (Jones, 2024). However, the risk of depoliticization and effacing of social inequalities even within these community yoga programs is present.

This article aims to contribute to this debate by first presenting the theoretical tensions around trauma and trauma-informed provision, the centrality of the body within the traumatization process, and the role of mind-body practices in addressing the impact of trauma. Then, we present the evaluation of the community yoga programs delivered to women with intersecting sociopolitical locations and complex needs. Although these services were not specifically directed to women with experience of interpersonal violence, service users belonged to groups subject to multiple intersecting oppressions, including domestic or sexual violence, homelessness, mental health issues, poverty, addiction, institutional racism, and strict immigration policies. As such, participants included women with experience of multiple forms of interpersonal and structural violences. By structural violence, we understand the type of violence that is embedded in sociocultural practices and institutions and operates invisibly and indirectly (Galtung, 1969). For the evaluation, we conducted interviews and body-mapping workshops with service users who had been attending the community yoga classes, to explore the perceived impact of yoga on the different dimensions (physical, emotional, social) of women's lives. The article discusses the results of the evaluation, as well as how the organization aimed to interweave the principles of compassionate embodied inquiry and intersectional feminism in both the delivery of the programs and the evaluation process itself.

Both the article and the evaluation project were developed collaboratively by a feminist researcher with extensive yoga practice (first author: ET) and two co-founders of a community yoga organization (second: MC and third author: LHR) based in the United Kingdom. We consider that synergies between academia and community organizations are necessary for the development of feminist theory and practice grounded in the lived experiences of women. Both our academic and community work are underpinned by an intersectional feminist framing of trauma, which acknowledges trauma as intrisically sociopolitical (Burstow, 2003, 2005) and embedded in interlocking power imbalances. We agree with Burstow (2003) that systemic oppression is a “traumatizing condition” (p. 1308) that interacts with the trauma experienced at interpersonal level. Overall, this paper adopts an intersectional feminist lens proposed by Black feminists such as Crenshaw (1991), Collins (2009) and hooks (2015).

## The Tensions Around Trauma and Trauma-Informed Services

Trauma is a contested concept that has been conceptualized through different angles giving rise to a tension between the medical and the sociopolitical framing of trauma (Stein et al., 2007). Feminists have criticized the medical model of trauma—which focuses on disorder diagnosis and symptomatology reduction—and urged for an exploration of the socio-cultural context producing traumatic experiences (Burstow, 2003, 2005; Herman, 1997). The medical framing of trauma was originally received positively by feminist scholars and clinicians, as it was considered useful for the naming of the serious harm produced by violence against women. Yet, the later development of trauma theory and practice was criticized for psychologizing social issues and not putting enough emphasis on the gendered configurations of power underpinning violence and abuse (Brown, 2004; Gilfus, 1999; Tseris, 2013, 2017). Herman's (1997) framing of women's traumatic experiences as qualitatively different, due to the ongoing and repetitive nature of domestic and childhood abuse, operated as a “bridge” between the psychiatric establishment and feminist politics (Tseris, 2017, p. 255). However, it could be argued that she did not distance herself from the disorder model and used misnomers such as Complex Posttraumatic Stress Disorder, which may indicate a *post* patriarchal society where abuse no longer exists (Burstow, 2005). This disorder may further obscure the imminent risk and everyday potentiality of violence against women (Vera-Gray, 2020). Although the acknowledgment of the traumatic impact of gender-based violence validated the harmful experience of women, it ended up pathologizing women and disregarding their resilience (Brown, 2004; Tseris, 2013). Furthermore, the lack of recognition of the strategies women use to navigate and enhance their lives when living with mental health issues is even more pronounced for Black women (Sosulski et al., 2010). Thiara and Harrison (2021) stated that pathologizing responses were more prominent towards minoritized women due to discriminatory attitudes within services, with this leading to “dual pathologizing” (p. 48). Overall, feminist critics of trauma acknowledge the potential benefits of the trauma framework but remain skeptical of its individualizing approach, which obscures the role of multiple oppressive structures in the traumatizing experience.

Although feminist thinking focused on trauma which was related to violence against women and was inscribed within the gendered arrangements of power, other types of systemic oppression were also acknowledged as conducive to traumatization (Brown, 1995; Burstow, 2003). Burstow (2003) discussed the harmful impact of oppressive structures which act in an invisible, and insidious way, drawing on the work of Brown (1995) and Root (1992). Insidious traumatization refers to the cumulative, ongoing but indirect traumatic experience of individuals immersed in oppressive structures such as sexism, racism, classism, ableism, and homophobia. Although the multilayered nature of trauma was articulated mainly by feminist scholarship to foreground the multiple and intersecting sites of oppression (as discussed above), psychiatric literature attempted to move beyond the individualistic approach acknowledging racial, gender, or class inequalities as potential compounding factors of complex traumatization (see Ford & Courtois, 2020). However, we agree with Tseris (2017) that sociopolitical issues are largely being erased within the mental health services provision, as evidence-based trauma therapies are limited in their capacity to address the structural conditions of oppression. This raises the question if community services are better positioned to be able to adopt a critical feminist framework.

Although community organizations acknowledge the points of contention around the apolitical nature of trauma “industry,” they still emphasize the relevance of trauma-informed services. In the UK, the inadequacy of mainstream services in understanding the complex realities of women at the intersection of multiple oppressions has strengthened calls for trauma-informed approaches (Holly, 2017; Thiara & Harrison, 2021). Specifically, Holly (2017) evidenced the need for the provision of trauma-informed services to women experiencing multiple disadvantages. This was aligned with other research showing the positive outcomes of a trauma-informed approach to the health and well-being of women with complex needs (Sharpen, 2018). Specifically in relation to the gender-based violence sector, Thiara and Harrison (2021) pointed to the relevance of trauma-informed services able to understand and respond to the complex traumatic experiences of Black and minoritized survivors of violence and abuse, the intergenerational trauma pertaining to historic racialized oppression. However, the recognition of socio-cultural issues and systemic oppressions is not always embedded in the framing of trauma-informed provision. Specifically in relation to the trauma approaches within the UK sexual violence sector, Vera-Gray (2020) discussed how the professionalization of the Rape Crisis centers has led to the deemphasizing of the social dimensions of sexual violence, the depoliticizing of the sector and, consequently, the risk of losing its original ethos. However, she acknowledged the value of trauma-informed approaches, with the condition that the societal dimensions of violence are addressed and the individual "goal of recovery" is being replaced by collective empowerment (p.68).

## Trauma and the Body

Over the last decades, there has been growing emphasis on the centrality of the body within the traumatic experience from different disciplinary perspectives. Neuroscientific studies have evidenced that trauma is encoded in the body, by producing neurophysiological changes, decreased somatic awareness and further dysregulation (Smith & Ford, 2020; Van der Kolk, 2015). For example, women who have experienced prolonged, cumulative trauma, such as childhood or domestic abuse may feel disconnected from their bodies (Herman, 1997). This neurobiological basis of trauma was met again with skepticism by feminist authors suggesting that the focus on neuroscience is reductionist and privileges the narrative on deficiencies over resilience resulting from trauma (Tseris, 2013). At the same time, it is worth noting that the neurobiological framings of trauma focused mainly on interpersonal violence. Nevertheless, other feminist thinkers such as Burstow (2003), who embraced a more holistic concept of trauma, linking this with structural oppression, acknowledged the *physical aspect* of all types of harm, including the non-bodily and more insidious ones. She commented on how trauma may make the individual feel alienated from their body, which resonated with neuroscientific literature on the dissociative impacts of trauma (Herman, 1997). Arguably the insidious nature of systemic oppression makes the somatic impact of trauma more invisible and buried under the skin. Overall, both interpersonal and sociocultural violence may have a somatic imprint which may be difficult to surface; while the somatic effect of explicit violence was evidenced through a neurobiological lens, less emphasis has been placed on the impact of systemic oppression on the body.

The somatic impact of oppression could be inscribed within the theoretical discussion around the effect of societal inequalities on the body (Shilling, 2012). More specifically, the embodied aspect of oppression was foregrounded by feminist, critical race, and embodiment theorists who argued that structural inequalities are enacted and perpetuated through bodies and their interaction with other bodies (Menakem, 2021; Ng, 2018; Young, 2005). For example, Ng (2018) suggested that unequal relationships of power are enacted in the body, in such a way that they are constantly being reproduced, even in the bodies of those who have learned to think critically about these injustices. Although a wider analysis of the relationship of the body with society is not within the scope of this article, it is worth noting the underlying theoretical tensions around the body's capacity to exercise agency within societal structures (Alaimo & Hekman, 2008; Shilling, 2012). We argue that it is important to reclaim the “voice” of our bodies, acknowledging their limitations associated with both biological conditions and societal structures, yet also acknowledging their capacity for change.

## Trauma and Mind–Body Practices

The focus on the centrality of the body led to the development of body-based methodologies. From a clinical perspective, body-based interventions were developed to address the trauma stored in the body and used either as stand-alone or complementary to talk-based interventions (Van der Kolk, 2015). The need to address the body as the locus of the traumatized self was also foregrounded because of the inability of a part of traumatized populations to engage in talk therapies (Smith & Ford, 2020). Although this body-centered framework reflected a move away from the dualistic mind-body approach, the research on the effectiveness of these mind-body practices is based on the medical model—disorder diagnosis and treatment for symptom reduction. This is again a reductionist approach to trauma that does not capture the multilayered nature of the traumatization process. Additionally, the focus on symptom reduction is dissonant with the holistic approach of mind-body practices which target the multiple dimensions of the trauma healing process (Niles et al., 2018). Conversely, from a decolonizing perspective, mind-body practices have been framed as strategies for critical embodied learning, using the body as a tool for personal and collective healing as well as resistance to societal oppressions (Batacharya & Wong, 2018; Ng, 2018). These perspectives move beyond the individualistic model of mind-body practices to a more politicized approach to the body. Batacharya (2018) argued that somatic therapies in the clinical field are insufficient if they do not promote a witnessing of both the self and its sociopolitical location.

Yoga is a mind-body practice that has been increasingly used in therapeutic settings to address the somatic impacts of trauma, with growing evidence demonstrating its effectiveness for improved self-regulation, capacity for self-care, and relationships with others (Rhodes, 2015; Rhodes et al., 2016; van der Kolk et al., 2014). Yoga includes physical postures, breathwork, and meditative practices, and has been used as an intervention for various physical and mental health conditions as well as general well-being (Niles et al., 2018). Its therapeutic application to address the negative effects of trauma came to prominence following the development of a trauma-sensitive yoga intervention (Emerson & Hopper, 2011). This trauma-informed approach is centered on the principles of *safety, empowerment* and *choice* and aims to enhance the somatic awareness and self-regulation of trauma survivors. Although the popularity of yoga as a therapeutic intervention for trauma has increased over the recent years, yoga teachers have been pointing to the need for a trauma-informed approach beyond the therapeutic settings; this approach has been considered important to accommodate the needs of people who have experience of trauma and marginalization and may not have access to other healing spaces (Wood, 2024). However, the race and class dynamics inherent in the mainstream yoga studios make them inaccessible to people from marginalized groups. Overall, modern yoga provision has been criticized for reinforcing social relations of power and being inscribed within neoliberal capitalist discourses and white supremacist logic (Batacharya, 2018; Jain, 2020; Lucia, 2020).

In an attempt to address the depoliticization of mainstream yoga, community yoga has sought to emphasize how personal healing is inextricably linked to social justice (Jones, 2024). Community yoga projects have been set up to increase the reach of yoga to wider society, improving access for populations who have been excluded from these practices, because they are unaffordable or are perceived to belong to specific privileged groups. Jones (2024) pointed out how these initiatives may be even more relevant since the COVID-19 pandemic, which has contributed significantly to the increase of mental health problems. As such, these projects have been trying to increase accessibility to spaces of healing for *diverse* bodies, which may not feel welcome in places that are dominated by female, white, thin, able-bodied, heterosexual, cis-gendered bodies covered in expensive yoga outfits. Furthermore, community yoga programs have attempted to challenge the cultural appropriation of yoga for New Age purposes, as well as acknowledge the historical roots of the practice, and how it has changed across different social and political contexts. However, these nonprofit yoga practices and discourses have also been also criticized as “mere gestures” of subversion to neoliberal spirituality (Jain, 2020, p. 168). Jain discussed specifically the gestural nature of “trauma-informed” projects offering yoga classes to prisoners, such as The Prison Yoga Project and the Africa Yoga Project; by promoting healing and individual transformation, they arguably end up sidelining structural factors leading to state violence and mass incarceration. Nevertheless, the author does not negate the possibility of more subversive spiritual practices and institutions, as long as these seek to promote self-care as a form of “embodied resistance” which is “when we love and uplift the deviant or divergent body, rather than burden it with the responsibility to recover or morally police it” (Jain, 2020, p. 170).

In the following section, our discussion revolves around an evaluation study of the services provided by a UK outreach community yoga organization. First, we present the service model and underpinning philosophy of the organization and then examine the methodology and findings of the evaluation conducted in 2021.

## The Case of a UK Community Yoga Organization

The organization was set up in 2017 by a group of yoga professionals (including the second and the third author) to offer trauma-informed yoga practices to disenfranchised communities who cannot access mainstream yoga provision. The founding of the organization was driven by the impulse to resist the appropriation of yoga by privileged normative bodies and raise the profile of societal issues permeating the local yoga community. It is important to note that the organizational ethos has also been guided by the yogic philosophical tenets of *daya* (compassion for the suffering in oneself and others) and *seva* (selfless service) alongside the concept of *svādhyāya* (self-inquiry). As such, the emphasis is placed on the provision of classes to underrepresented populations and the fostering of a critical self-inquiry ethos—both within the organization and across the sector—rooted in embodied knowing. Critical self-reflection is foregrounded as core to the organizational praxis and ethos; through this lens, yoga professionals can examine their social location of power and privilege and reflect on how this may shape their teaching and how they relate to others (both students and other people in the yoga community). Moreover, the organization acknowledges that oppressions are carried in the body, and a trauma-informed lens is important when delivering classes to marginalized populations; safety, choice and agency over body movement are the main considerations of trauma-informed yoga provision (Emerson & Hopper, 2011; McAtee and Wilson, 2024). Under this trauma-informed approach, yoga professionals need to be aware of how people's lives may be affected by both individual and systemic trauma, as well as how to support individuals in community yoga spaces without compounding the trauma carried in them. Overall, the organization is actively seeking to challenge the hegemonic narratives of yoga as a white, middle-class, cis-gendered women-dominated space and thus address the inequities in access to these trauma-informed practices, as potential pathways to healing. The organization's founders have promoted this interweaving of the trauma lens and ongoing scrutiny of societal issues. MC is a clinical psychologist who specializes in complex trauma and shares with the team her clinical knowledge around the physiological, emotional and social impacts of trauma; LHR has a strong community work background, weaving in an activist and systemic lens which supports the organizational praxis and reflective practices of the team members.

In praxis, the commitment to dismantling these inequities and opening space for diverse bodies to practice together, is operationalized through different ways: bespoke classes for marginalized populations in their community settings; diversity of the teaching team, in terms of race, ethnicity, disability and gender identity; “check-in,” “check-out” spaces at the beginning and end of the classes as spaces of reflection and connection, possibility for yoga students to take ownership of their practice by making requests and offering feedback at the end. Overall, this service model aims to elicit feelings of connectedness and reciprocity, among service users and between students and teachers. Participants are not usually aware that the service model aims to be trauma-informed and socially engaged; and this may operate as a barrier to engagement, as evidenced by our research. Additionally, in line with the trauma-informed service model, the organization is committed to a collaborative model, working alongside service users (Elliott et al., 2005).

## The Evaluation Study and Methodological Approach

In 2021, the authors conducted a study to evaluate the impact of the outreach yoga organization. This responded to the need to understand the outcomes of their work and potential areas of improvement as well as understand “what matters” to yoga students. Additionally, in line with a trauma-informed service model based on the principles of collaboration and empowerment, the evaluation aimed to foreground the voices and needs of service users, enabling them to contribute to future service provision. We also anticipated that the process could provide a space for yoga students to reflect on their embodied experiences and how these may be related to other spheres of their lives, offering them the opportunity to attune to their embodiment with curiosity and acceptance. Thus, we aimed to bring the compassionate embodied knowing, embedded in the yoga practice, into the evaluation process itself. This could be considered a trauma-informed research method, in which participants are called to reflect on their lives by fostering empathy for their own embodied experience and that of others (Brigden, 2022).

Feminist principles informed the study's methodological approach (Skinner et al., 2005; Westmarland & Bows, 2018). These approaches foreground the voices of women and other oppressed groups, acknowledge the power imbalances within the research process, and aim to produce knowledge that may improve the material realities of women's lives. Moreover, we drew on the value of embodiedexperience, in line with feminist approaches considering “women as embodied subjects who think, act and know through their bodies” (Davis, 2007, p. 57). As such, we aimed to unveil the knowing which emerges through the interweaving of the physical, affective, cognitive, and socio-cultural aspects of women's embodied experiences. We also agree with Davis (2007) that the embodied knowing -which may surface after being previously suppressed- may be more easily integrated into a political agenda. The importance of embodied experience as a starting point for exploring intersectional oppressions has also been discussed by scholars who bring a decolonial feminist perspective to knowledge production (Batacharya & Wong, 2018; Ng, 2018). This epistemological emphasis on the body as a source of knowledge informed our data collection tools—described in the next section—but also our reflexive stance as researchers. Although reflexivity is a crucial tenet of feminist research, we agree with Petillo and Hlavka (2022) that the sentient realm of the researcher's reflexivity needs to be further explored. If, as Ng (2018) suggested, “relationships of power are never enacted merely in the form of intellectual encounters” but “entail a confrontation of bodies, which are differently inscribed” (pp. 35–36), could we, as researchers, afford to disregard our sensations during the reflexivity process? At the same time, the foregrounding of the embodied experience is interwoven with the ethics of care, which is one of the critical aspects of Black feminist epistemology (Collins, 2009). Collins articulated the value placed on emotional expressiveness, empathy and connectedness as part of Black feminist knowledge production. As yoga practitioners, we consider that embodied knowing and attunement to our feelings and sensations enable us to feel more connected with our interlocutors. As Petillo and Hlavka (2022) note, unlike an objective observer who cannot be attuned to their feelings, “an embodied, entangled observer is invested and honors awareness and honesty with interlocutors” (p. 23). We believe that the intersectional feminist approaches that informed our methodological choices, were also aligned with the philosophical tenets of yoga embedded in the organizational ethos (compassion and self-study), as described above. In the next section, we will describe how our epistemological stance and emphasis on embodied knowing and self-reflexivity, shaped our research process.

## Methods and Participants

For the research process, we used interviews and body mapping workshops to explore the various dimensions in which participants may have experienced the impact of yoga. We aimed to understand any potential changes in their physical, mental, and emotional states, as well as their relationships with others and the world around them. We were also interested to hear their views about how we could measure the benefits of yoga in an accessible and inclusive way. Enquiry into appropriate outcomes measurement methods was core to the project, as it enabled participants to contribute to future organizational practice that attends to their needs. However, in this article, we will focus the discussion on students’ views on their experiences with yoga. Two body mapping workshops and six interviews were conducted from July to September 2021. The study participants were service users and staff members of two local partner organizations, providing services to women experiencing multiple oppressions (including gender violence, racism, poverty, and homelessness). Some of the women also experienced mental health and substance abuse issues. The participants were service users who had been attending the yoga programs delivered by the community yoga organization (both online and in-person). Ten yoga students and a support worker (who provided translation services) attended the in-person workshops; four yoga students and two staff members were interviewed. Two yoga students attended both the body mapping workshop and the interview. The study was conducted while the COVID-19 pandemic was still unfolding and restrictions were partially in place.

Body mapping is an arts-based method “positioned at the intersection of research, community development and art therapy” (Collings et al., 2021, p. 57). For our body mapping workshops, we used a pre-drawn life-size body and asked participants to portray changes they had experienced when practicing yoga using different art materials, including coloring pencils, scissors, stickers, post-it notes, tissue paper, and objects from nature (e.g., leaves). More specifically, we asked them to think about how they have experienced the impact of yoga in the physical body as well as the emotional, mental, and social spheres of their lives. We offered prompts to facilitate the process, suggesting they could use the different body parts as symbols (e.g., the hands for relationships with others or the world, the heart for emotions, the head for thoughts and mental processes). The participants used the same body map to depict their experiences. The second and third authors delivered the workshops, and the first author attended via video conferencing, recording the sessions and taking field notes. At the start of the workshops, we used a “check-in” activity, which involved all participants – including the facilitators – sharing one or two words about their sentient state. We explicitly stated that participants could nod or not share anything with the group, if they preferred. Then, we moved to a table where the body map was placed, and we explained the process. We repeatedly reassured the participants that the activity was for them to reflect on and depict their experiences, and that artistic skills were not required. Participants had time to reflect on the questions and engage with the materials at their own pace. As they were all working with the same body map, they could interact with each other and comment on each other's contributions. After this, they were asked to comment on the visual or textual elements they added to the map. The activity ended with a “check-out” process, where participants and facilitators could express their feelings at that moment.

As the study aimed to capture the potential impact of yoga across the different dimensions of women's lives, we considered that the embodied inquiry offered by the body mapping would be suitable for our research. This method focuses on embodied experience and facilitates the exploration of both the sensory and social aspects of embodiment (Boydell, 2021). As such, it aligns well with the embodied awareness aspect of the yoga programs and our methodological focus on embodied knowing. Additionally, body mapping enables participants to express their experiences non-verbally in a way that is appropriate and accessible to them, thus facilitating the participation of people who may not feel able to articulate their experiences due to language barriers (Gastaldo et al., 2012). This strengthened the appropriateness of the method, as over half of our participants were not native English speakers. Moreover, we considered that body mapping could be a valuable tool for our study as it can help participants express emotions which are held in the body and are difficult to articulate (Collings et al., 2022). For the interviews, we did not use a body mapping method, but we still tried to elicit the affective dimensions of participants’ experiences using cards with printed images. Similarly to the “check-in,” “check-out” process of the workshops, both interviewers and interviewees expressed their sentient state by choosing a card. Participants were also offered the option to reply to the interview questions by choosing an image that reflected visually their perceptions and views. The cards were available for the rest of the interview, and participants were reminded that they were welcome to use them to support their reflection on the question.

Regarding recruitment of participants, yoga students were identified via the two partner organizations. Those who had attended more than three yoga classes were informed about the project by the partner organization and invited to participate in a workshop or an individual interview. One staff member from each partner organization was also identified and invited to participate. Service users from the same organization attended the same workshop. Overall, we recruited 13 yoga students and three staff members. In terms of the demographic profile of participants, data was available for 11 yoga students. All of them identified as women, six were Black African, one was Asian, three were white, and one unknown ethnicity. Regarding the age group, seven were between 27 and 35 years old, three were 40 and 50 years old, and one was over 55. In relation to religion, seven identified as Muslim, one as spiritual, two with no religious identity, and one unknown. None of the participants identified as disabled. Most participants had no prior experience of yoga before joining the community classes.

In line with feminist evaluation processes focusing on reciprocity, we allocated part of our funding to cover the participants’ travel expenses and offer them vouchers for their contribution. Hague & Mullender (2005) clearly stated the importance of “making the giving two-way, so that women participating also receive” and acknowledging their participation as remunerated work, rather than expecting women “to participate in their spare time and out of kindness, goodwill, or personal commitment” (p. 158). A support worker who attended the workshop also provided translation services when needed. For the workshops, we chose community spaces that participants had used before, and aimed to create an emotionally safe and caring environment, offering drinks and snacks. We also arranged for a support worker to be available during the workshops, to ensure participants could access emotional support and manage potential uncomfortable feelings. Hague and Mullender (2005) also discussed the importance of creating a safe space and not compounding the trauma, when consulting women who experienced abuse. This is consistent with the trauma-informed lens and ethics of care, embedded in the yoga organization and informed the evaluation process itself. Relatedly, we considered that the participation of the facilitators in the “check-in,” “check-out,” and card activities enabled connectedness and reciprocity.

At the first stage of the research, we conducted the two body mapping workshops, which generated visual and verbal data from participants’ body maps and verbal narratives. For the analysis of the workshops, the first author transcribed the discussions and took pictures of the body maps. Following familiarization with the data, ET compared the visual elements that each participant incorporated in the collective body map with their verbal narratives to avoid misinterpreting the meanings participants attributed to their visual contributions. Visual elements were interrogated in terms of their semantic content and their repetition and positioning in the map (what part of the body, in the space inside or outside of the body). Through concurrent visual and verbal data coding, ET identified initial themes and then discussed them with MC and LHR for further refinement. The most salient themes relating to the benefits of yoga practice informed the interviews—which were conducted after the workshops—to ensure that they were relevant for participants. Following the completion of the interviews, ET transcribed the data and through iterative engagement with the data, coded the data set and identified initial patterns of meaning. These were presented to the other two authors, and following discussion and comparison with the initial themes of the workshops, the final themes were defined. MC and LHR brought in a clearer perspective of the group dynamics, as they were the ones who facilitated them. Overall, we followed a process of inductive thematic analysis from a critical-realist perspective (Braun & Clarke, 2006, 2014). Through this approach, we aimed to understand how women made meaning of their embodied experiences and how the wider socio-cultural context shaped these experiences.

## Findings and Discussion

This section will present the findings and discussion of the themes which were identified throughanalysis of the visual and verbal data described above. Our initial themes were structured around the views of women about the benefits of yoga, which were aligned with the aim of the evaluation. These themes related to increased emotional regulation and capacity for self-care, improved sense of physical and emotional well-being, as well as connection with others and openness to the world, in line with the trauma healing model of Herman (1997). However, the analysis also identified how their narratives around their yoga experiences were shaped by their sociocultural identities, and as such elicited their initial perceptions of *otherness* within the yoga spaces. Furthermore, we noted that participants discussed aspects of their lives to express the process of change felt during the yoga programs. This allowed us to understand better the material conditions of their lives and the impact of individual and systemic trauma on health and wellbeing. Although this was not part of our initial aims of the study, it provided rich information to understand the context of students’ lives and needs in more depth. Moreover, we were able to understand how different intersecting identities shaped the experiences of trauma, healing, and well-being. All participants had experienced systemic trauma, yet their different socio-political locations brought nuances in the way they felt the benefits of yoga.

### The Othered Body

The first theme reflects women's feelings of otherness shaped by dominant yoga narratives. Participants (including all staff members and some of the service users) shared how preconceptions of yoga as a practice meant for specific bodies, contributed to a sense of lack of belonging in the yoga space. Issues of ethnic, cultural, and religious identity, socioeconomic location, body type, and counterculture status were perceived as markers of the yogic identity. It is also important to note that these perceptions differed according to the participants’ intersecting identities.

In the body mapping workshop in which nearly all women were Black African and Muslim, the staff member, who was involved in promoting the activities to service users, described how yoga is perceived as belonging to Indian or Hindu communities and is not appropriate for people coming from other religious or ethnic backgrounds.

… because it's still … there's a lot of women who do not practice yoga, because they heard so much about it, that it's not good … to do with … you know religious stuff or you know. […] You always kind of think that it's their thing … Asians, Indians. That belongs to them. You can never think of a dark person or another ethnicity doing it. You might think he's gone a bit funny. Hippies were fine doing it but seeing a black person, you could hardly connect that with a person doing yoga. (Somya)

This last quote suggests that even though yoga is perceived to be a culturally specific practice originating in South Asia, its use by “hippies” has been normalised. The same participant explained further that yoga is currently more associated with Western bodies rather than Asian ones and is still not representative of Black communities. It is identifiable through commodities, such as the yoga mat, and adopted by white, slim bodies. This is evidenced in the following interaction between two participants of the body mapping workshop:

Somya: We still think it's a Western thing, especially if I am talking about Black people, it's still a new thing for us … who does yoga and other activities as well …

Maryam : I thought it was for posh people before. Only posh people do yoga, that's what I thought …

Somya: Getting the carpet, the rag …

Maryam: Yeah, yeah I am going to do yoga … (laughing).

Relatedly, Jade, another staff member emphasized socioeconomic location as the main marker of identity producing feelings of otherness in yogic spaces. She stated how the perceptions of “yoga being a middle-class thing, something you know that's not for me … or like that's a hippy thing” may have shaped attitudes towards the practice. This was also expressed by Lucy, a white participant who described her initial resistance to engage with yoga:

I did used to think (laughing) … there I go … I did used to think all this stuff is airy fairy, I’m gonna be honest, I did … totally honestly, I used to think … oh god … do we have to do this? (Lucy)

Although some participants represented yogic symbols such as the “third eye” and the mantra “aum” on the body map, they unveiled their hesitancy in engaging with these symbols, arguably due to their association with New Age counterculture narratives. It was white participants who discussed the spiritual aspects of yoga, such as how the practice enabled them to "connect with something bigger". Although there was some resistance to engaging with the spiritual discourse and symbols of the yogic practice, they felt more confident to discuss their feelings of spiritual connection. Arguably, this may be related to the fact that they felt less bound by the discourses around yoga’s cultural and religious essentialism.

Overall, yoga seemed to enable participants to engage more critically with dominant discourses of “who belongs to yoga” and start challenging essentialist notions of yogic identity. These findings are aligned with the study of Batacharya (2018), who examined the yoga experiences of South Asian women in Canada. Although our study participants seemed to be less familiar with the discourses around the cultural appropriation of yoga—perhaps due to their different sociocultural location (mainly Black African and white)—they reflected critically on the exclusionary nature of yoga. They considered that current yoga practices are associated to affluent white groups and are non-inclusive of Black or Muslim people.

### Reclaiming a Sense of Healing and Well-Being

Most women brought a narrative aspect to their accounts, discussing the physical and mental health issues they were enduring prior to and during the yoga programs, how yoga gradually provided some relief and helped them reclaim a growing sense of healing and well-being -both physical and emotional. We noted that the positive impact of yoga was manifested slightly differently for the two groups of service users, potentially due to the different backgrounds of victimization. Overall, although only two participants referred explicitly to experiences of trauma, most of them described how their bodies and minds were suffering without naming experiences of traumatization or violence.

The participants from Black and ethnically minoritized communities discussed the impact of yoga on both their physical and mental well-being and, consequently, their improved sense of agency. They mentioned how the context of the COVID-19 pandemic and the associated lockdowns had contributed to the worsening of their health conditions, as well as an increased sense of isolation and loss of community. This may be due to the impact that COVID-19 had on these communities, exacerbating other types of structural violence, such as racialized and gendered violence, and limiting access to support routes (Thiara & Harrison, 2021; Thiara & Roy, 2022). In their narratives, participants from these communities described problems with sleep, back pain, digestive issues, as well as anxiety and depression; yoga was perceived as an opportunity to address these health conditions and improve their well-being.

We have had quite a hard time through lockdown … and we had stress and anxiety, we feel like we don’t know what we are having tomorrow … we cannot go outside … Yoga was an alternative to do, to do exercise, to get rid of tiredness, because at that time, we don’t have anything, we have a kind of depression […] When I start doing yoga, I finish it and I feel active and positive energy. It helped get rid of all the negative thinking. I feel different after yoga and helped me getting more stronger, like a change in our mood, we wake up, we have our breakfast, after we exercise in the yoga, we start thinking like we can go for a walk we can do something more. (Samira)

The focus on physicality, namely the impact of yoga on the physical body, was depicted on the body map of the specific group; the words “strength,” “flexibility,” and “energy” were written on different parts of the body and the intestine was drawn to represent relief from their digestive problems. One participant also drew a red line circulating around the body to depict the perceived changes in blood circulation. Overall, women shared that yoga practice made them feel “stronger,” “more active,” “confident,” and “motivated” to engage with life as well as practice self-care. Maryam also described this positive outlook on life:

I struggle with sleeping and stress and anxiety and stuff, so when I started doing yoga, I don’t know how to explain, the first day I did yoga it was so cold I slept … and after I felt I want to go outside … at that part of my life, I didn’t want to go outside. I just wanted to be in my room, I wasn’t even opening the window at that time, that's why I took the counselling with you, I didn’t want to open the window, I was so scared, lockdown stressed me and everything and yoga changed my life in a good way … I don’t want to cry …, when I was doing yoga, I was looking forward you know. (Maryam)

Regarding the participants who had histories of complex trauma (both individual and systemic) but were not racialized, their narratives of trauma and engagement with yoga focused more on their need to address their mental health issues. As such, for them, yoga was perceived more as a trauma-healing and safety-inducing practice rather than a physical well-being practice. Jade, the staff member of the organization who supported them, described how yoga could help women in their healing in a profound way, underlining safe embodiment as a trauma-informed aspect of the practice:

Yoga is hugely helpful in terms of supporting women to find safety in the body and to start experience enjoyment in being in the body, which is part of trauma-informed practice and matters for profound form of healing rather than just fixing … like learning a few tricks […] and I think something about the holding of the yoga class and the gentle pace of it can allow people to maybe just touch into … even maybe moments of inhabiting their bodies in way that feels safe, safe enough, and I think that feels really profound. (Jade)

This emphasis on improved emotional regulation was also shared by the service users of this organization. They discussed how the yogic routine enabled them to feel an increased sense of control over their trauma symptoms, bodies and lives. Three participants discussed the use of breathing and grounding techniques learnt in yoga to manage states of emotional dysregulation. Lucy discussed how the end part of the practice helped her connect with a deep state of rest and relaxation and overcome racing thinking:

I think it's the most fully relaxed I have ever felt apart from when I am asleep. Even then, I am kind of … jump at anything [showing with her face expression and hands a state of being hyperalert]. That made me realize I can get properly relaxed, which I never really believed. (Lucy)

The contribution of yoga to feeling “calm,” “relaxed,” “grounded,” and “present in my feet” was manifested in their group body map, with words and symbols—reflecting the feeling of groundedness—placed across different parts of the body.

Overall, the narratives of women with complex needs indicated that their yoga practice contributed to a sense of relief from the impact of trauma on their bodies, while participants from Black and ethnic minorities articulated an increased sense of physical and emotional well-being since they started practicing yoga. The group of ethnically minoritized women discussed how yoga helped them with their physical ailments and improved their emotional state during the COVID-19 pandemic, when the community on-line yoga classes were the only space of self-care and agency for these women. This need for alternative health practices has already been pointed out by Thiara and Harrison (2021); women from minoritised communities are less likely to engage with mainstream health services and value a holistic offer of services, including complementary therapies like yoga. The group of women who were not racialized but had complex needs, discussed more the benefits of yoga for emotional regulation and increased capacity to stay calm and grounded. The practice represented a way to address the impact of trauma in everyday life; although they discussed the effects of trauma on their bodies, they did not comment on the societal causes of their trauma.

### Acceptance and Connection to Others

This theme relates to the two concepts that sustained and enabled the process of change activated through the yoga practice; an attitude of acceptance towards the limitations of their present conditions and a feeling of being held and connected to others around them. While the attitude of acceptance was not clearly articulated by participants, it was manifested in the way they experienced their journey, the openness to negotiate their expectations from the practice and accept the process of change. Aisha, a Muslim woman, commented that she joined the classes intending to lose weight, but she ended up appreciating the relaxation benefits even though they did not match her initial purpose. Even though this readjustment did not indicate a process of body acceptance, it could be argued that it enabled her to shift attention from gendered body ideals to sentient embodiment; drawing on the spatial metaphor of Ng (2018), this could be a shift from the “outside-in” (p.34), the discursive constructions of the body, to the “inside-out,” (Batacharya and Wong, 2018, p.10) the body felt from inside. Moreover, this may also indicate that the fitness ideals of modern yoga which may govern people's initial engagement with the practice, can be replaced by others more aligned with their healing needs.

Other participants expressed their acceptance of the limitations and challenges that yoga presented for them when they started practicing. Rachel commented how she was initially self-conscious but gradually became more accepting of her body’s limitations.

I used to be self-conscious about my body a lot and how I looked when I was doing this. I guess this is why I wasn’t (going) to classes, because my brain was going, oh my god, I do not look like that, what's going on, but slowly, slowly I am getting better and I am (laughing, opening her arms) who cares? (Rachel)

Similarly, other participants across the two groups shared how they found difficult specific parts of the practice (such as the relaxation or specific poses) but they gradually noticed that the practice was getting easier. Jade explained the role of yoga in facilitating this attitude of acceptance:

Yoga as a practice that is really about bringing curiosity and being in your body, and being with what's there and bringing acceptance and noticing resistance. I don’t know... something about that feels really different and quite radical. (Jade)

It is interesting to note how resistance is framed negatively and in juxtaposition with acceptance, whereas from a liberatory practices perspective, resistance would be necessary for societal change to happen. However, the use of the term here refers to the internal resistance to change; noticing resistance is a way of witnessing the trauma carried in the body, as a necessary condition for transformation. Jade also discussed how this attitude of acceptance was fostered through the caring approach to teaching. This gentle and caring aspect of yoga promotes acceptance of the process as well enables the feeling of togetherness. All interviewees and participants from the two body mapping groups, mentioned the importance of sharing the practice with other women, with some of them emphasizing the aspects of fun and laughter interwoven into the practice. Another participant, Helen, appreciated the sense of belonging that yoga brought to her and also expressed feelings of embodied connection, “unity” with other students.

(Yoga) connects you with everybody in the room, at that moment and time, and that's important especially when you feel lonely in your life and you don’t have friends. And at that moment in time, you are with others.. the girls and there are boundaries, so that's why you feel safe also because there are boundaries … and at the same time with boundaries, we all feel together and that's beautiful as well. (Helen)

The sense of belonging was noted as an important factor to dissipate the *felt* otherness experienced by women, when first considering joining the yoga classes. Practicing yoga with other people from the same community shifted these thoughts and enabled the gradual dissipation of the feelings of otherness and turned fear of being judged to feelings of belonging in the group.

They have never done yoga before, they were scared because in a lot of culture they don’t know yoga, they think yoga is all about … kind of going to another laye[r] … you know … It got different meanings, they were really scared of doing it. (The yoga program) gave them the opportunity doing it with others of the same community, other women who have never done it before and built up that confidence … that was really important to do it together. (Somya)

We could argue that the yoga programs offered the space to challenge the *othering* yogic discourses and be part of a community, as well as afforded women the opportunity to regain a degree of agency, diminished by structural inequalities. Additionally, the improved sense of self and agency that emerged through yoga practice expanded women's space for action. Participants felt eager to take action not only to improve their own lives but these of their communities. This is evidenced by the expressed desire of some women to teach the yoga practice to other women, as discussed by a staff member of the organization working with minoritized women:

They want to change their lives, I know some of them started working and some of them they said they want to do yoga professionally, to teach someone as well … They asked about that … they need to know about how to deal with their bodies to show someone how to do it without hurting themselves. (Joud)

However, one could argue that this reclaiming of agency is not sufficient and does not constitute an act of resistance to social structures. We agree with Lugones (2003), as cited by Davis (2007) that more attention is needed to the subtle and multidirectional ways of women resisting oppressive structures; arguably increased capacity for self-care is a strategy of resistance. We argue that the need for structural change cannot disregard the need for reclaiming space for healing and well-being. Practicing self-care and fostering an attitude of acceptance and compassion towards the limitations of our bodies and material lives is necessary to the structural change needed; albeit we are clear that this is not sufficient. We think that the concept of radical self-care, as proposed by Lorde (2017), needs to be brought to the fore of our praxis, both as practitioners and researchers, and guide us how to be more attentive to this interlacing between the “eternal ordinary” and the life-changing, “the mundane and the apocalyptic” (p. 130). Self-care is necessary to attend to the woundedness that systemic and individual trauma has brought into our bodies. We agree with Haines (2019) about the importance for social movements to increase awareness of how trauma is imprinted on the body and shapes our survival strategies. And this can be done safely within an ethical framework of care and compassion as shown by feminist epistemologies (Collins, 2009).

## Conclusion

In this article, we examined the problematic issues around trauma narratives and yoga practices, and drew into the findings of our evaluation study to explore if and how trauma-informed community yoga programs could represent a life-enhancing option for women who experience multiple and interlocking systems of oppressions. We also interrogated the liberatory potential of the community programs exploring their capacity to foster critical examination and potential resistance to social hierarchies. Our evaluation process mirrored the delivery of the trauma-informed yoga practice in community settings in two different aspects: focusing on the body as a site of knowledge by using a body mapping method which fosters a more embodied and sentient approach to knowledge production; and incorporating an ethical framework of care and reciprocity. This embodied epistemic approach was appreciated by the participants as it created a relational and creative space and offered more time for self-reflection. Analysis showed that yoga facilitated critical inquiry of the social and cultural identities related to yoga, and to some extent, enabled participants to challenge the exclusionary aspect of modern yoga spaces. When practicing with other women with similar cultural backgrounds, women felt able to challenge the feeling of otherness in the yogic space. At the same time, women reflected on their health conditions and the impact of trauma in their lives.Although the evaluation aimed to explore the broader benefits of the practices rather than focus on their trauma healing potential, women discussed how yoga helped them alleviate the impact of trauma and improve their sense of wellbeing. Overall the community yoga programs promoted the creation of embodied relational spaces; these communities of practice and affect enabled women to enter a space of healing and well-being that was not initially perceived as theirs.

In terms of the potential role of these yoga programs to be conducive to social change, it is important to acknowledge that participants presented yoga as a self-care or healing tool, and not as a space of resistance to social structures. One could argue that the focus on individual well-being, which was enhanced through the yoga programs, was more aligned with New Age discourses and could have obscured how structural inequalities shaped health conditions (Batacharya, 2018). While their participation in the classes may have presented them the opportunity to acknowledge dominant neoliberal yoga narratives, it did not offer the space for deep exploration of issues of systemic injustices. However, these community yoga programs offered women the space to connect with their embodied self and reappropriate their body's capacity for change. As Federici (2020) points out, the “reappropriation of our body” and its agency, becomes more urgent in a world, where the body is represented as a “source of epidemics” and a “repository of diseases” (p. 123). We agree with Owens (2020), that embodiment is “the most radical act because there is no liberation without the union of body and mind” (p. 119). Additionally, we argue that holding community spaces which promote embodied self-inquiry, curiosity, and acceptance, can and needs to pave the way for creating communities of resistance. We understand that for an organizational practice to be liberatory it is important to have an ongoing practice of embodied self-inquiry as well as ethics of care and reciprocity. As Haines (2019) suggested, social organizations need to understand how the somatic imprint of trauma (individual and collective) may shape unhelpful responses within social movements. Thus, we consider that the attention of community yoga organizations to embodied self-inquiry and ethics of compassion and care, informed by the interweaving of yoga philosophy, intersectional feminist theory and the trauma-informed lens can facilitate transformation at personal and potentially systemic level.
